# Dr. Cameron, on the Tincture of Coccinella

**Published:** 1801-06

**Authors:** Ch. Cameron

**Affiliations:** Worcester


### *To the Editors of the Medical and Phyfical Journal.*

GENTLEMEN,

AMONG many important purpofes your ufeful publication is calculated to ferve, not the leaft is, that it affords a refuge to minute and detached hints and obfervations, which, though not of fufficient confequence to be ufhered into the world in a feparate publication, yet by their accumulation, may affift in promoting the improvement of medical fcience. Under this impreffion, permit me to offer you a trifle, which, if it will not take take up too much room, to the exclusion of more important communications, you may, perhaps, favour with a place in your next Journal.

Some time ago, Dr. Beddoes recommended as a cure for the tooth-ach, to bruise a *lady-cow* between the fingers, and apply them to the aching tooth, and the experiment was, I believe, often attended with the desired effect. The insect meant I take to be the coccinella septem punctata of Linnæus. But as those animals are not to be had at all seasons of the year, it occurred to me that a tincture of them might probably answer the same purpose as the fresh insect; and there happening to be a great abundance of them last summer, I desired the apothecary to our Infirmary to make a saturated tincture of them in proof spirit, which has proved of great service in several instances of tooth-ach: But the circumstance to which I wish principally to draw the attention of your readers is this; that the tinctura coccinellæ, in colour, smell, and taste, very nearly resembles a tincture of opium. If it should also turn out to have similar, or any other, good effects when given internally, it may be a fortunate discovery; but on this head I regret my inability to say any thing at present, as the bottle in which it was made was unfortunately broken, and only a small quantity of it saved. This hint, however, will, I dare say, be sufficient to induce many of your readers to make it, and try its virtues; and should any beneficial consequences ensue, the purpose of this letter will be fully answered to,

